# Psychological Issues among Adult Patients with Untreated Clef Lip and Palate: Algerian Survey-based study

**DOI:** 10.1192/j.eurpsy.2023.847

**Published:** 2023-07-19

**Authors:** M. A. Rais, A. Moussa

**Affiliations:** 1 Department of Dentistry, Faculty of Medicine of Algiers, Algiers; 2 Faculty of Medicine, University of Blida, Blida, Algeria

## Abstract

**Introduction:**

Cleft lip and palate are birth defects that occur when a baby’s lip or mouth do not form properly during pregnancy. Individuals with a cleft of the lip and/or palate (CL/P) differ from their peers due to their facial appearance, hearing and speech problems, in addition, it causes psychosocial impacts among affected children. However, among untreated adults, it seems that psychological sequelae can be major and life limiting.

**Objectives:**

The purpose of this study is to assess Algerian untreated young CL/P patients psychological and lifestyle issues.

**Methods:**

This is a survey-based cross-sectional study conducted during August- October 2022 in Algeria through an online survey which is a self-administered questionnaire ( SAQ). The study target population were young individuals ( age between 17 and 30) with untreated or partially treated clef lip and palate confitions. The questionnaire consisted of 10 multiple questions including a demographic section, evaluating patients satisfaction, lifestyle, psychological problems related to their conditions and limitations due to it. All participants provided their informed consent prior to their participation. The Statistical Package for the Social Sciences (SPSS) version 22.0 was used to analyse the collected data.

**Results:**

Final sample size consisted in 207 responses. The mean age of the participants was 24.3± 3 years. Findings showed that slightly more that three fourths (75,9%) of participants reported that they are unsatisfied of their life because of their CL/P condition. Among 120 uneducated participants, (67%) of them reported thay they abandoned school because of their appearance. For P <0.001, suicide thoughts were higher among female participants (38%) than male participants (9%). Almost half of respondents (49.4%) presented strong immigration desires due to their education, relationships and psychosocial health issues.

**Conclusions:**

The psychological impact of cleft lip and palate in adults appears to be disturbing, chronic and life-limiting. Urgent approaches to ensure the early management of cleft lip and palate in Algeria remain mandatory. Increasing the number of specialized maxillofacial services seems to be a good first step.

**Disclosure of Interest:**

None Declared

